# 5-Phenyl-3-(2-thien­yl)-1,2,4-triazolo[3,4-*a*]isoquinoline

**DOI:** 10.1107/S1600536810003181

**Published:** 2010-01-30

**Authors:** F. Nawaz Khan, P. Manivel, K. Prabakaran, Venkatesha R. Hathwar, Seik Weng Ng

**Affiliations:** a Chemistry Division, School of Science and Humanities, VIT University, Vellore 632 014, Tamil Nadu, India; b Solid State and Structural Chemistry Unit, Indian Institute of Science, Bangalore 560 012, Karnataka, India; c Department of Chemistry, University of Malaya, 50603 Kuala Lumpur, Malaysia

## Abstract

In the title mol­ecule, C_20_H_13_N_3_S, the triazoloisoquinoline ring system is approximately planar, with an r.m.s. deviation of 0.045 Å and a maximum deviation of 0.090 (2) Å from the mean plane for the triazole ring C atom which is bonded to the thio­phene ring. The phenyl ring is twisted by 52.0 (1)° with respect to the mean plane of the triazoloisoquinoline ring system. The thio­phene ring is rotationally disordered by approximately 180° over two sites, the ratio of refined occupancies being 0.73 (1):0.27 (1).

## Related literature

For the synthesis and anti­helmintic activity of triazolo compounds similar to the title compound, see: Nadkarni *et al.* (2001 ▶).
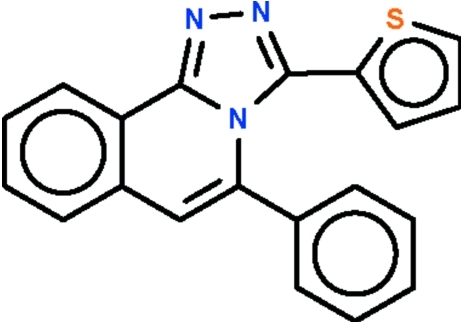


## Experimental

### 

#### Crystal data

C_20_H_13_N_3_S
*M* *_r_* = 327.39
Orthorhombic, 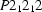

*a* = 19.7715 (17) Å
*b* = 8.7735 (7) Å
*c* = 9.3027 (8) Å
*V* = 1613.7 (2) Å^3^
*Z* = 4
Mo *K*α radiation
μ = 0.21 mm^−1^
*T* = 293 K
0.32 × 0.30 × 0.24 mm

#### Data collection

Bruker SMART area-detector diffractometer
Absorption correction: multi-scan (*SADABS*; Sheldrick, 1996 ▶) *T*_min_ = 0.937, *T*_max_ = 0.952
10809 measured reflections
3670 independent reflections
2414 reflections with *I* > 2σ(*I*)
*R*_int_ = 0.045

#### Refinement

*R*[*F* ^2^ > 2σ(*F* ^2^)] = 0.057
*wR*(*F* ^2^) = 0.128
*S* = 1.03
3670 reflections
230 parameters
45 restraints
H-atom parameters constrained
Δρ_max_ = 0.20 e Å^−3^
Δρ_min_ = −0.21 e Å^−3^
Absolute structure: Flack (1983 ▶), 1631 Friedel pairs
Flack parameter: 0.05 (13)

### 

Data collection: *SMART* (Bruker, 2004 ▶); cell refinement: *SAINT* (Bruker, 2004 ▶); data reduction: *SAINT*; program(s) used to solve structure: *SHELXS97* (Sheldrick, 2008 ▶); program(s) used to refine structure: *SHELXL97* (Sheldrick, 2008 ▶); molecular graphics: *X-SEED* (Barbour, 2001 ▶); software used to prepare material for publication: *publCIF* (Westrip, 2010 ▶).

## Supplementary Material

Crystal structure: contains datablocks global, I. DOI: 10.1107/S1600536810003181/lh2984sup1.cif

Structure factors: contains datablocks I. DOI: 10.1107/S1600536810003181/lh2984Isup2.hkl

Additional supplementary materials:  crystallographic information; 3D view; checkCIF report

## Figures and Tables

**Fig. 1.**
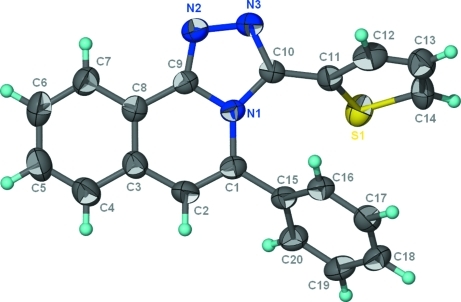
Thermal ellipsoid plot (Barbour, 2001) of C20H13N3S at the 50% probability level; hydrogen atoms are drawn as spheres of arbitrary radius. The disorder is not shown.

## supplementary crystallographic information

### Comment

The molecular structure of the title compound is shown in Fig. 1.

### Experimental

2-(3-Phenylisoquinolin-1-yl)hydrazine (1 mmol) was condensed with thienyl-2-carbaldehye (1.1 mmol) under refluxing conditions isopropanol (10 ml) solvent to give the corresponding 2-(3-phenylisoquinolin-1-yl)-1-(2-thienylmethylene)hydrazine in high yield. The compound was then oxidatively cyclized in nitrobenzene (10 ml) at 473 K. The product was recrystallized from dichlomethane to give block-shaped crystals.

### Refinement

Hydrogen atoms were placed in calculated positions (C–H 0.93 Å) and were included in the refinement in the riding model approximation, with *U*(H) set to 1.2*U*(C).

The thienyl ring is disordered over two positions. The temperature factors of the primed atoms were restrained to those of the unprimed ones, and the anisotropic temperature factors were restrained to be nearly isotropic. Pairs of distances of the primed atoms were restrained to within 0.01 Å of the umprimed ones.

### Crystal data

**Table:**

C_20_H_13_N_3_S	*F*(000) = 680
*M**_r_* = 327.39	*D*_x_ = 1.348 Mg m^−^^3^
Orthorhombic, *P*2_1_2_1_2	Mo *K*α radiation, λ = 0.71073 Å
Hall symbol: P 2 2ab	Cell parameters from 1886 reflections
*a* = 19.7715 (17) Å	θ = 2.4–20.1°
*b* = 8.7735 (7) Å	µ = 0.21 mm^−^^1^
*c* = 9.3027 (8) Å	*T* = 293 K
*V* = 1613.7 (2) Å^3^	Block, yellow
*Z* = 4	0.32 × 0.30 × 0.24 mm

### Data collection

**Table:**

Bruker SMART area-detector diffractometer	3670 independent reflections
Radiation source: fine-focus sealed tube	2414 reflections with *I* > 2σ(*I*)
graphite	*R*_int_ = 0.045
φ and ω scans	θ_max_ = 27.5°, θ_min_ = 2.1°
Absorption correction: multi-scan (*SADABS*; Sheldrick, 1996)	*h* = −25→25
*T*_min_ = 0.937, *T*_max_ = 0.952	*k* = −11→10
10809 measured reflections	*l* = −10→12

### Refinement

**Table:**

Refinement on *F*^2^	Secondary atom site location: difference Fourier map
Least-squares matrix: full	Hydrogen site location: inferred from neighbouring sites
*R*[*F*^2^ > 2σ(*F*^2^)] = 0.057	H-atom parameters constrained
*wR*(*F*^2^) = 0.128	*w* = 1/[σ^2^(*F*_o_^2^) + (0.0577*P*)^2^] where *P* = (*F*_o_^2^ + 2*F*_c_^2^)/3
*S* = 1.03	(Δ/σ)_max_ = 0.001
3670 reflections	Δρ_max_ = 0.20 e Å^−^^3^
230 parameters	Δρ_min_ = −0.21 e Å^−^^3^
45 restraints	Absolute structure: Flack (1983), 1631 Friedel pairs
Primary atom site location: structure-invariant direct methods	Flack parameter: 0.05 (13)

### Fractional atomic coordinates and isotropic or equivalent isotropic displacement parameters (Å^2^)

**Table:**

	*x*	*y*	*z*	*U*_iso_*/*U*_eq_	Occ. (<1)
S1	0.59650 (8)	0.46180 (17)	0.63475 (18)	0.0639 (5)	0.731 (3)
S1'	0.5970 (3)	0.1599 (5)	0.7573 (7)	0.0639 (5)	0.27
N1	0.50872 (10)	0.2300 (3)	0.3908 (2)	0.0425 (6)	
N2	0.41393 (12)	0.1323 (3)	0.4794 (3)	0.0563 (7)	
N3	0.45695 (12)	0.1669 (3)	0.5908 (3)	0.0555 (7)	
C1	0.55374 (13)	0.2743 (3)	0.2800 (3)	0.0468 (7)	
C2	0.52959 (14)	0.2706 (4)	0.1445 (3)	0.0583 (8)	
H2	0.5581	0.3016	0.0707	0.070*	
C3	0.46266 (14)	0.2218 (4)	0.1071 (3)	0.0563 (8)	
C4	0.43864 (17)	0.2247 (5)	−0.0345 (4)	0.0718 (10)	
H4	0.4665	0.2600	−0.1078	0.086*	
C5	0.37500 (18)	0.1765 (4)	−0.0660 (4)	0.0746 (11)	
H5	0.3598	0.1784	−0.1607	0.090*	
C6	0.33259 (17)	0.1246 (4)	0.0419 (4)	0.0715 (10)	
H6	0.2891	0.0924	0.0193	0.086*	
C7	0.35420 (15)	0.1203 (4)	0.1817 (4)	0.0615 (9)	
H7	0.3254	0.0858	0.2538	0.074*	
C8	0.41991 (14)	0.1682 (3)	0.2156 (3)	0.0483 (7)	
C9	0.44535 (13)	0.1724 (3)	0.3605 (3)	0.0449 (7)	
C10	0.51257 (13)	0.2250 (3)	0.5399 (3)	0.0448 (7)	
C11	0.56696 (13)	0.2788 (3)	0.6329 (3)	0.0492 (7)	
C12	0.5985 (3)	0.2087 (6)	0.7424 (7)	0.0665 (19)	0.731 (3)
H12	0.5866	0.1084	0.7623	0.080*	0.731 (3)
C13	0.6458 (4)	0.2730 (7)	0.8243 (11)	0.0695 (18)	0.731 (3)
H13	0.6711	0.2269	0.8965	0.083*	0.731 (3)
C14	0.6491 (3)	0.4187 (7)	0.7808 (6)	0.0649 (19)	0.731 (3)
H14	0.6770	0.4906	0.8243	0.078*	0.731 (3)
C12'	0.5862 (10)	0.4243 (14)	0.659 (2)	0.0665 (19)	0.27
H12'	0.5619	0.5062	0.6221	0.080*	0.269 (3)
C13'	0.6415 (10)	0.447 (3)	0.740 (2)	0.0695 (18)	0.27
H13'	0.6697	0.5313	0.7463	0.083*	0.269 (3)
C14'	0.6445 (15)	0.3135 (19)	0.811 (3)	0.0649 (19)	0.27
H14B	0.6729	0.3042	0.8903	0.078*	0.269 (3)
C15	0.62471 (13)	0.3176 (3)	0.3121 (3)	0.0460 (7)	
C16	0.66749 (13)	0.2237 (3)	0.3895 (3)	0.0491 (7)	
H16	0.6519	0.1311	0.4251	0.059*	
C17	0.73382 (14)	0.2682 (4)	0.4135 (3)	0.0565 (8)	
H17	0.7625	0.2051	0.4657	0.068*	
C18	0.75759 (15)	0.4039 (4)	0.3615 (4)	0.0634 (9)	
H18	0.8019	0.4337	0.3797	0.076*	
C19	0.71543 (15)	0.4960 (4)	0.2821 (4)	0.0659 (9)	
H19	0.7315	0.5875	0.2449	0.079*	
C20	0.64940 (14)	0.4525 (4)	0.2576 (3)	0.0569 (8)	
H20	0.6212	0.5150	0.2036	0.068*	

### Atomic displacement parameters (Å^2^)

**Table:**

	*U*^11^	*U*^22^	*U*^33^	*U*^12^	*U*^13^	*U*^23^
S1	0.0616 (8)	0.0510 (8)	0.0790 (10)	−0.0019 (6)	−0.0066 (7)	−0.0081 (7)
S1'	0.0616 (8)	0.0510 (8)	0.0790 (10)	−0.0019 (6)	−0.0066 (7)	−0.0081 (7)
N1	0.0383 (11)	0.0443 (14)	0.0448 (14)	0.0009 (10)	0.0050 (10)	0.0028 (11)
N2	0.0483 (14)	0.0649 (16)	0.0557 (16)	−0.0058 (12)	0.0043 (13)	0.0079 (13)
N3	0.0510 (14)	0.0667 (17)	0.0489 (15)	−0.0028 (13)	0.0059 (12)	0.0061 (13)
C1	0.0424 (14)	0.0469 (15)	0.0511 (18)	0.0034 (13)	0.0072 (13)	0.0010 (15)
C2	0.0534 (17)	0.077 (2)	0.0448 (18)	−0.0024 (16)	0.0093 (14)	0.0053 (17)
C3	0.0519 (17)	0.067 (2)	0.0497 (19)	0.0066 (15)	−0.0018 (14)	−0.0057 (16)
C4	0.064 (2)	0.100 (3)	0.051 (2)	0.009 (2)	0.0022 (17)	−0.004 (2)
C5	0.067 (2)	0.101 (3)	0.056 (2)	0.014 (2)	−0.0124 (18)	−0.015 (2)
C6	0.056 (2)	0.078 (2)	0.080 (3)	0.0023 (18)	−0.018 (2)	−0.014 (2)
C7	0.0504 (18)	0.067 (2)	0.067 (2)	−0.0026 (16)	−0.0045 (15)	0.0007 (17)
C8	0.0467 (16)	0.0454 (16)	0.0529 (18)	0.0058 (13)	0.0007 (14)	−0.0047 (14)
C9	0.0413 (14)	0.0432 (16)	0.0501 (17)	−0.0003 (12)	0.0024 (14)	0.0033 (14)
C10	0.0472 (15)	0.0431 (16)	0.0440 (17)	0.0028 (13)	0.0045 (13)	0.0023 (14)
C11	0.0463 (15)	0.0544 (17)	0.0470 (17)	0.0081 (13)	0.0031 (13)	−0.0061 (15)
C12	0.073 (3)	0.045 (3)	0.082 (4)	0.002 (3)	0.012 (3)	0.011 (3)
C13	0.054 (3)	0.091 (4)	0.064 (3)	0.015 (4)	−0.004 (2)	−0.002 (4)
C14	0.063 (3)	0.068 (4)	0.063 (4)	0.005 (3)	−0.019 (3)	−0.021 (3)
C12'	0.073 (3)	0.045 (3)	0.082 (4)	0.002 (3)	0.012 (3)	0.011 (3)
C13'	0.054 (3)	0.091 (4)	0.064 (3)	0.015 (4)	−0.004 (2)	−0.002 (4)
C14'	0.063 (3)	0.068 (4)	0.063 (4)	0.005 (3)	−0.019 (3)	−0.021 (3)
C15	0.0441 (14)	0.0470 (17)	0.0471 (17)	−0.0013 (13)	0.0073 (13)	−0.0016 (13)
C16	0.0470 (15)	0.0484 (16)	0.0520 (18)	0.0027 (14)	0.0057 (14)	0.0051 (14)
C17	0.0451 (15)	0.069 (2)	0.0555 (18)	0.0142 (16)	0.0029 (13)	−0.0004 (17)
C18	0.0441 (17)	0.080 (2)	0.066 (2)	−0.0092 (16)	0.0086 (16)	−0.0034 (19)
C19	0.0613 (19)	0.063 (2)	0.074 (2)	−0.0157 (17)	0.0121 (17)	0.0096 (18)
C20	0.0518 (17)	0.0544 (18)	0.064 (2)	−0.0016 (15)	0.0031 (15)	0.0112 (16)

### Geometric parameters (Å, °)

**Table:**

S1—C11	1.708 (3)	C10—C11	1.459 (4)
S1—C14	1.752 (4)	C11—C12	1.344 (5)
S1'—C11	1.667 (5)	C11—C12'	1.355 (8)
S1'—C14'	1.717 (9)	C12—C13	1.331 (6)
N1—C9	1.380 (3)	C12—H12	0.9300
N1—C10	1.389 (3)	C13—C14	1.342 (6)
N1—C1	1.417 (3)	C13—H13	0.9300
N2—C9	1.316 (4)	C14—H14	0.9300
N2—N3	1.375 (3)	C12'—C13'	1.343 (8)
N3—C10	1.301 (3)	C12'—H12'	0.9300
C1—C2	1.348 (4)	C13'—C14'	1.342 (8)
C1—C15	1.484 (4)	C13'—H13'	0.9300
C2—C3	1.434 (4)	C14'—H14B	0.9300
C2—H2	0.9300	C15—C20	1.377 (4)
C3—C8	1.398 (4)	C15—C16	1.383 (4)
C3—C4	1.400 (4)	C16—C17	1.387 (4)
C4—C5	1.359 (5)	C16—H16	0.9300
C4—H4	0.9300	C17—C18	1.368 (4)
C5—C6	1.385 (5)	C17—H17	0.9300
C5—H5	0.9300	C18—C19	1.376 (4)
C6—C7	1.369 (5)	C18—H18	0.9300
C6—H6	0.9300	C19—C20	1.379 (4)
C7—C8	1.402 (4)	C19—H19	0.9300
C7—H7	0.9300	C20—H20	0.9300
C8—C9	1.439 (4)		
			
C11—S1—C14	90.5 (2)	C10—C11—S1'	118.2 (3)
C11—S1'—C14'	84.6 (8)	C12—C11—S1	105.3 (3)
C9—N1—C10	104.0 (2)	C10—C11—S1	124.2 (2)
C9—N1—C1	121.5 (2)	S1'—C11—S1	117.3 (2)
C10—N1—C1	134.5 (2)	C13—C12—C11	124.5 (5)
C9—N2—N3	106.4 (2)	C13—C12—H12	117.8
C10—N3—N2	109.6 (2)	C11—C12—H12	117.8
C2—C1—N1	116.8 (2)	C12—C13—C14	105.4 (6)
C2—C1—C15	122.0 (3)	C12—C13—H13	127.3
N1—C1—C15	121.2 (2)	C14—C13—H13	127.3
C1—C2—C3	124.2 (3)	C13—C14—S1	114.2 (5)
C1—C2—H2	117.9	C13—C14—H14	122.9
C3—C2—H2	117.9	S1—C14—H14	122.9
C8—C3—C4	118.7 (3)	C13'—C12'—C11	118.0 (17)
C8—C3—C2	118.9 (3)	C13'—C12'—H12'	121.0
C4—C3—C2	122.4 (3)	C11—C12'—H12'	121.0
C5—C4—C3	120.8 (3)	C14'—C13'—C12'	100.6 (19)
C5—C4—H4	119.6	C14'—C13'—H13'	129.7
C3—C4—H4	119.6	C12'—C13'—H13'	129.7
C4—C5—C6	120.4 (3)	C13'—C14'—S1'	121.1 (17)
C4—C5—H5	119.8	C13'—C14'—H14B	119.4
C6—C5—H5	119.8	S1'—C14'—H14B	119.4
C7—C6—C5	120.6 (3)	C20—C15—C16	119.1 (3)
C7—C6—H6	119.7	C20—C15—C1	118.8 (3)
C5—C6—H6	119.7	C16—C15—C1	122.0 (3)
C6—C7—C8	119.7 (3)	C15—C16—C17	119.6 (3)
C6—C7—H7	120.2	C15—C16—H16	120.2
C8—C7—H7	120.2	C17—C16—H16	120.2
C3—C8—C7	119.9 (3)	C18—C17—C16	120.9 (3)
C3—C8—C9	117.2 (2)	C18—C17—H17	119.6
C7—C8—C9	122.8 (3)	C16—C17—H17	119.6
N2—C9—N1	110.8 (3)	C17—C18—C19	119.5 (3)
N2—C9—C8	127.9 (2)	C17—C18—H18	120.2
N1—C9—C8	121.2 (3)	C19—C18—H18	120.2
N3—C10—N1	109.2 (2)	C18—C19—C20	120.0 (3)
N3—C10—C11	122.3 (2)	C18—C19—H19	120.0
N1—C10—C11	128.5 (2)	C20—C19—H19	120.0
C12—C11—C12'	99.4 (9)	C19—C20—C15	120.9 (3)
C12—C11—C10	130.1 (3)	C19—C20—H20	119.6
C12'—C11—C10	128.3 (9)	C15—C20—H20	119.6
C12'—C11—S1'	111.4 (9)		
			
C9—N2—N3—C10	−0.4 (3)	N1—C10—C11—C12'	69.1 (13)
C9—N1—C1—C2	5.9 (4)	N3—C10—C11—S1'	53.0 (4)
C10—N1—C1—C2	−176.4 (3)	N1—C10—C11—S1'	−129.4 (4)
C9—N1—C1—C15	−172.2 (2)	N3—C10—C11—S1	−120.4 (3)
C10—N1—C1—C15	5.5 (5)	N1—C10—C11—S1	57.2 (4)
N1—C1—C2—C3	−1.5 (5)	C14'—S1'—C11—C12	−2(3)
C15—C1—C2—C3	176.5 (3)	C14'—S1'—C11—C12'	−10.5 (17)
C1—C2—C3—C8	−2.8 (5)	C14'—S1'—C11—C10	−175.0 (13)
C1—C2—C3—C4	177.7 (4)	C14'—S1'—C11—S1	−1.1 (13)
C8—C3—C4—C5	0.0 (5)	C14—S1—C11—C12	1.2 (5)
C2—C3—C4—C5	179.6 (3)	C14—S1—C11—C12'	58 (5)
C3—C4—C5—C6	0.5 (6)	C14—S1—C11—C10	174.5 (3)
C4—C5—C6—C7	−0.3 (6)	C14—S1—C11—S1'	1.0 (4)
C5—C6—C7—C8	−0.3 (5)	C12'—C11—C12—C13	−12.8 (12)
C4—C3—C8—C7	−0.6 (5)	C10—C11—C12—C13	−176.7 (7)
C2—C3—C8—C7	179.8 (3)	S1'—C11—C12—C13	175 (3)
C4—C3—C8—C9	−177.6 (3)	S1—C11—C12—C13	−4.0 (9)
C2—C3—C8—C9	2.8 (4)	C11—C12—C13—C14	4.9 (11)
C6—C7—C8—C3	0.8 (5)	C12—C13—C14—S1	−3.3 (8)
C6—C7—C8—C9	177.6 (3)	C11—S1—C14—C13	1.3 (5)
N3—N2—C9—N1	1.2 (3)	C12—C11—C12'—C13'	21 (2)
N3—N2—C9—C8	−175.8 (3)	C10—C11—C12'—C13'	−174.8 (14)
C10—N1—C9—N2	−1.4 (3)	S1'—C11—C12'—C13'	23 (2)
C1—N1—C9—N2	176.9 (2)	S1—C11—C12'—C13'	−104 (6)
C10—N1—C9—C8	175.8 (3)	C11—C12'—C13'—C14'	−22 (3)
C1—N1—C9—C8	−5.9 (4)	C12'—C13'—C14'—S1'	13 (3)
C3—C8—C9—N2	178.0 (3)	C11—S1'—C14'—C13'	−2(3)
C7—C8—C9—N2	1.2 (5)	C2—C1—C15—C20	52.5 (4)
C3—C8—C9—N1	1.3 (4)	N1—C1—C15—C20	−129.5 (3)
C7—C8—C9—N1	−175.5 (3)	C2—C1—C15—C16	−124.6 (4)
N2—N3—C10—N1	−0.5 (3)	N1—C1—C15—C16	53.4 (4)
N2—N3—C10—C11	177.5 (3)	C20—C15—C16—C17	1.4 (4)
C9—N1—C10—N3	1.2 (3)	C1—C15—C16—C17	178.5 (3)
C1—N1—C10—N3	−176.9 (3)	C15—C16—C17—C18	−0.1 (4)
C9—N1—C10—C11	−176.6 (3)	C16—C17—C18—C19	−1.2 (5)
C1—N1—C10—C11	5.3 (5)	C17—C18—C19—C20	1.1 (5)
N3—C10—C11—C12	51.1 (6)	C18—C19—C20—C15	0.2 (5)
N1—C10—C11—C12	−131.3 (5)	C16—C15—C20—C19	−1.5 (4)
N3—C10—C11—C12'	−108.5 (13)	C1—C15—C20—C19	−178.7 (3)
